# Supplementary material for: Genomic Tools for the Characterization of Local Animal Genetic Resources: Application in Mascaruna Goat
Source: Animals (Basel). 2022 Oct 19;12(20):2840. doi: 10.3390/ani12202840 (PMC9597745; doi:10.3390/ani12202840)
Supplement: Supplementary file 1 [file animals-12-02840-s001.zip › Figure S3.pdf]

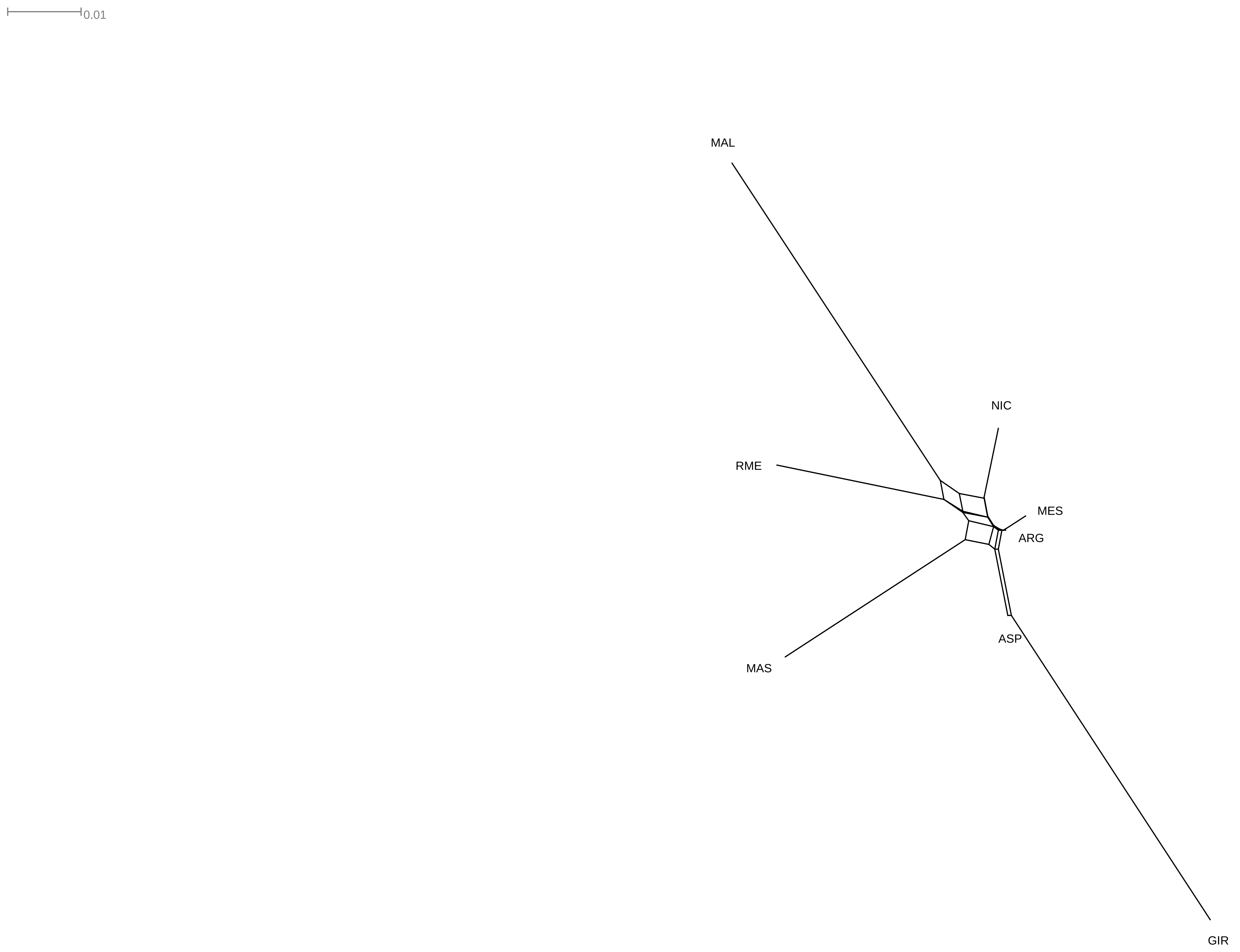
0.01
MAL
NIC
RME
MES
ARG
ASP
MAS
GIR
