# Supplementary figures and images for: Genomic Tools for the Characterization of Local Animal Genetic Resources: Application in Mascaruna Goat
Source: Animals (Basel). 2022 Oct 19;12(20):2840. doi: 10.3390/ani12202840 (PMC9597745; doi:10.3390/ani12202840)

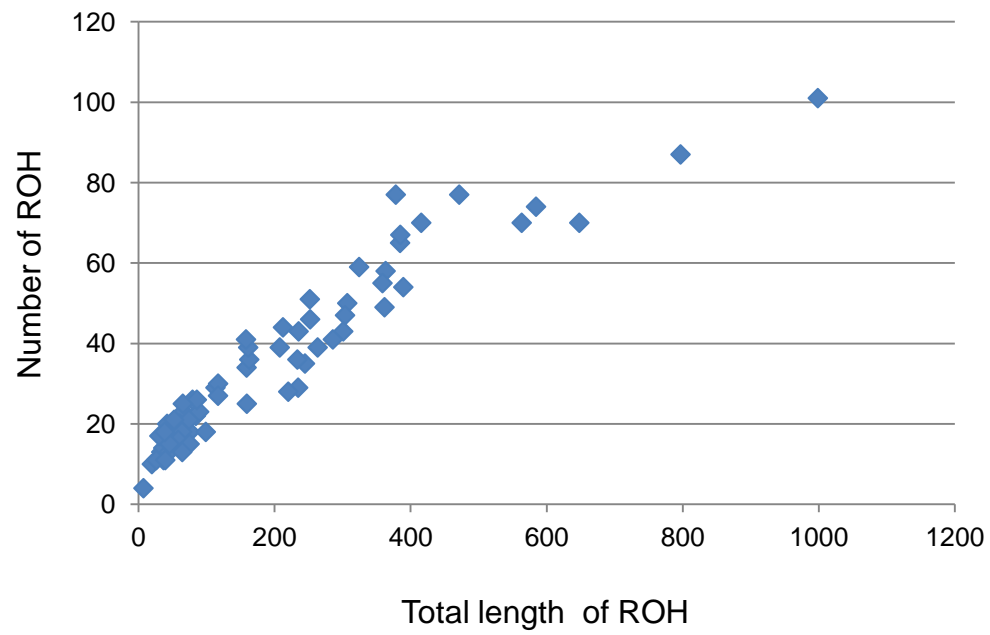

Supplement: Supplementary file 1 [file animals-12-02840-s001.zip › Figure S1.pdf]

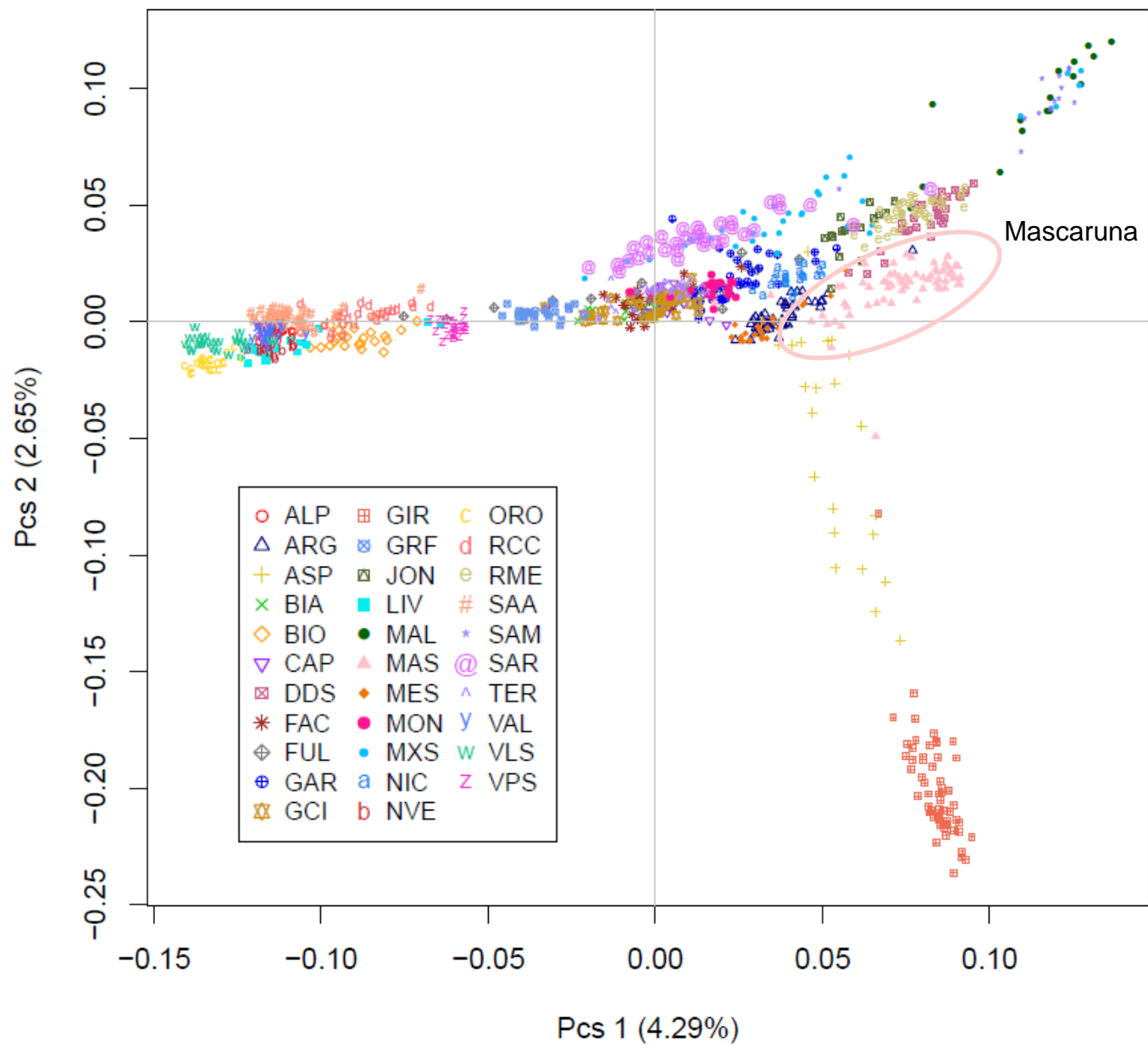

Supplement: Supplementary file 1 [file animals-12-02840-s001.zip › Figure S2.pdf]

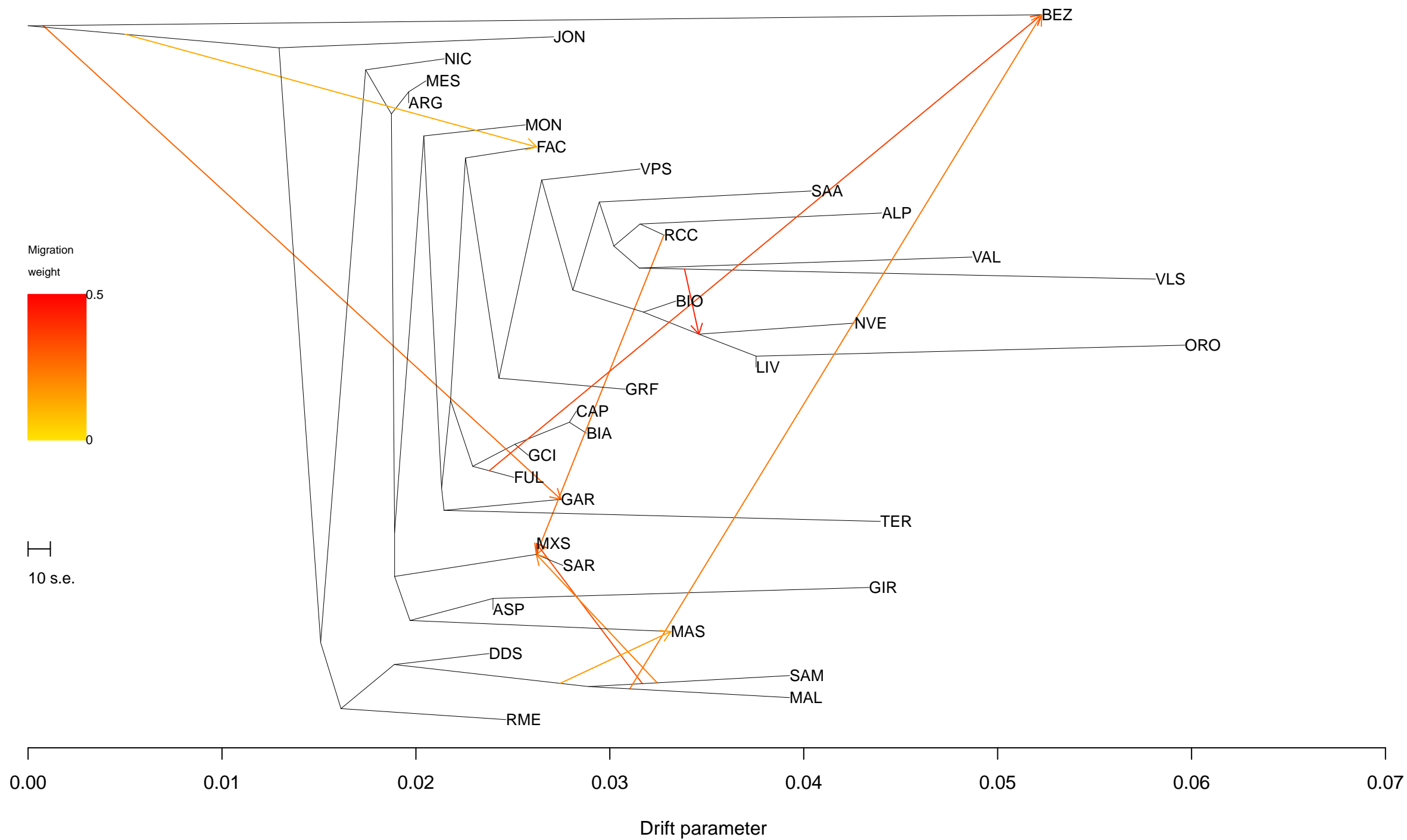

Supplement: Supplementary file 1 [file animals-12-02840-s001.zip › Figure S5.pdf]
